# Developmental exposure to near roadway pollution produces behavioral phenotypes relevant to neurodevelopmental disorders in juvenile rats

**DOI:** 10.1038/s41398-020-00978-0

**Published:** 2020-08-17

**Authors:** Elizabeth L. Berg, Lauren R. Pedersen, Michael C. Pride, Stela P. Petkova, Kelley T. Patten, Anthony E. Valenzuela, Christopher Wallis, Keith J. Bein, Anthony Wexler, Pamela J. Lein, Jill L. Silverman

**Affiliations:** 1 MIND Institute and Department of Psychiatry and Behavioral Sciences, University of California Davis School of Medicine, Sacramento, CA USA; 2 Department of Molecular Biosciences, University of California Davis School of Veterinary Medicine, Davis, CA USA; 3 Air Quality Research Center, University of California Davis, Davis, CA USA

**Keywords:** Psychiatric disorders, Learning and memory

## Abstract

Epidemiological studies consistently implicate traffic-related air pollution (TRAP) and/or proximity to heavily trafficked roads as risk factors for developmental delays and neurodevelopmental disorders (NDDs); however, there are limited preclinical data demonstrating a causal relationship. To test the effects of TRAP, pregnant rat dams were transported to a vivarium adjacent to a major freeway tunnel system in northern California where they were exposed to TRAP drawn directly from the face of the tunnel or filtered air (FA). Offspring remained housed under the exposure condition into which they were born and were tested in a variety of behavioral assays between postnatal day 4 and 50. To assess the effects of near roadway exposure, offspring of dams housed in a standard research vivarium were tested at the laboratory. An additional group of dams was transported halfway to the facility and then back to the laboratory to control for the effect of potential transport stress. Near roadway exposure delayed growth and development of psychomotor reflexes and elicited abnormal activity in open field locomotion. Near roadway exposure also reduced isolation-induced 40-kHz pup ultrasonic vocalizations, with the TRAP group having the lowest number of call emissions. TRAP affected some components of social communication, evidenced by reduced neonatal pup ultrasonic calling and altered juvenile reciprocal social interactions. These findings confirm that living in close proximity to highly trafficked roadways during early life alters neurodevelopment.

## Introduction

Neurodevelopmental disorders (NDDs) result from abnormal brain development and include a wide range of conditions, such as intellectual disability, attention deficit hyperactivity disorder (ADHD), and autism spectrum disorder (ASD). Symptoms present in early childhood and persist throughout life, significantly affecting social, cognitive, and behavioral functioning. ASD and ADHD, which affect ~1 and 5% of children respectively, are among the most common and well-studied NDDs^1^. The disorders often co-occur with 30–50% of ASD patients presenting symptoms of ADHD, and their prevalence is on the rise^2^. According to the U.S. Centers for Disease Control and Prevention, ASD is currently estimated to affect about 1 in 59 children, which represents a dramatic increase from their previously reported 1 in 68 estimate^3,4^. These increased prevalence rates highlight the crucial need to develop a better understanding of the etiology of these neurological disorders since these conditions already incur immense societal and economic costs.

While there is compelling evidence that susceptibility to NDDs, as well as symptom severity and treatment outcomes, are influenced by the interaction of genetic and environmental risk factors, the underlying mechanisms remain to be elucidated^4–7^. Environmental factors also contribute to these conditions—although researchers disagree on the relative contributions of genes and environment. Furthermore, studies suggest that more than 50% of new ASD cases are due to factors other than diagnostic drift^7–12^.

Identifying and understanding the environmental risk factors contributing to the rising prevalence rates is crucial and important since they can be modified and/or avoided, unlike genetic risk factors, which are, for the most part, not currently modifiable risk variables. Mounting epidemiological data using independent samples, models, and methods from a variety of geographical locations have implicated exposure to traffic-related air pollution (TRAP) as one of these factors. Human exposure to TRAP and/or proximity to roadways, especially during the late gestational period and/or early life, has been significantly associated with an NDD diagnosis^13–33^.

These studies of humans, however, fall short of establishing a causal relationship between TRAP exposure and NDD development, due to an array of confounding factors and a lack of data quantifying individual exposures to complex environmental mixtures. Animal models, therefore, offer a unique benefit and can be used to fill this knowledge gap and directly test the hypothesis that exposure to TRAP impairs behaviors related to NDDs (e.g., developmental delays, social interaction, learning and memory). While there has been some concentrated research in preclinical models, many of the commonly employed exposure methods are limited in their translational relevance to the human condition due to reasons such as repeated anesthesia and failure to recapitulate the complexity and/or relative concentrations of traffic-related emissions in the real world^34–39^.

In order to fully understand the behavioral consequences of near roadway exposures during early life, we leveraged an innovative real-time rodent exposure facility to expose developing rats. Since composition, dose, duration, intensity, mixtures, and timing of air pollution exposures can influence biological outcomes^40–42^, we designed our study to be translationally relevant by representing human TRAP exposure and combined real-world composition of pollutants and dosing in an animal model. The detailed components of the exposure can be found in our Supplementary Information and are reported in comprehensive detail in Bein et al. (under review)^43^. In brief, air from a traffic tunnel in northern California was diverted to a nearby exposure facility housing a large rat colony, with half the colony receiving polluted tunnel air and half the colony receiving filtered air. Using rats, which possess a larger and more sophisticated repertoire of social behaviors compared to mice, allowed for an extensive and nuanced examination of NDD-relevant outcomes. After delivering real-world polluted air to pregnant rats and their offspring, we sought to determine whether the gestational and early life near roadway exposure affected physical growth, neonatal reflexes, communication, social interaction, and/or learning and memory, using a battery of validated behavioral assays.

Numerous epidemiological studies have associated near roadway exposure to a range of diseases, but it is difficult in such studies to disentangle confounders, such as socioeconomic status, smoking, and diet. The Childhood Autism Risks from Genetics and Environment (CHARGE) study examined the link between autism and living near freeways using a distribution of distances: closest 10% (<309 m), next 15% (309–647 m), the next 25% (647–1419 m), and farthest from freeways (>1419 m)^28,44^. In each trimester of pregnancy, living closest (<309 m) to the freeway was associated with autism, with the odds ratio reaching the highest significance during the third trimester, informing the timing of our exposure period. In order to generate toxicological data that complements the epidemiological data, the exposure facility that we employed in this study was designed to model near roadway exposures of air pollution, noise, and vibration, the same stressors experienced by people living in this environment. Epidemiological studies differ on the distance from the roadway that is “safe”, as this distance is partially determined by how much the air pollution from vehicles dilutes and how much the noise and vibration dissipates before the near roadway population is exposed. We drew air from the eastern face of the tunnel, not from inside the tunnel itself, so that the air pollution was somewhat diluted already, we insulated the building to reduce noise, and we installed vibration isolators on the feet of the exposure chambers to reduce vibration. The goal of these measures was to expose the rodents to conditions that well model human exposures.

Our investigation led to the discovery that gestational and early life exposure to TRAP affects some components of social communication. Importantly, we also discovered that both roadside-reared groups, TRAP and filtered air (FA), with exposure to the same noise and vibrational stress, had significantly delayed growth and development of psychomotor reflexes, displayed altered social interactions, and exhibited abnormal motor activity. Histological outcomes from these exposures are described in our companion manuscript^45^. Further, we found no evidence for an effect, due to stress or otherwise, of the pregnant dams’ transport to the roadside facility on offspring behavior. This is the first report of functional outcomes of this exposure model, and the first report that illustrates behavioral deficits resulting from near roadway exposure alone.

## Methods

### Subjects

All animals were housed in a temperature-controlled vivarium maintained on a 12:12 light–dark cycle. All procedures were approved by the Institutional Animal Care and Use Committee (IACUC) of the University of California Davis (UC Davis) and were conducted in accordance with the National Institutes of Health Guide for the Care and Use of Laboratory Animals. To identify rats, pups were labeled with paw tattoos on postnatal day (PND) 2 using non-toxic animal tattoo ink (Ketchum Manufacturing Inc., Brockville, ON, Canada). Ink was delivered into the center of the paw with a 23-gauge hypodermic needle tip. Rats were also tail marked with non-toxic permanent marker at weaning to allow for additional identification. The tattoo and tail marks for each subject were coded to allow investigators to carry out testing and scoring blind to treatment group.

### Order of behavioral testing and description of cohorts

Male and female Sprague-Dawley rat breeders (PND 80–90) were paired for two weeks before females were singly housed at approximately gestational day (GD) 14. A group of dams was transported to the roadside exposure facility adjacent to a major freeway tunnel system in the Bay Area of Northern California. Dams were randomly assigned to one of two exposure conditions within the same facility: traffic-related air pollution (TRAP) or filtered air (FA). Two male and two female offspring from each of 20 dams were tested as follows: (1) developmental milestones at PND 4, 6, 7, 9, 10, and 12, (2) pup USV at PND 5, (3) reciprocal social interaction at PND 32–34, (4) open-field behavior at PND 39–41, (5) novel object recognition at PND 40–42, and (6) fear conditioning at PND 44–48.

A second group of dams remained housed at a UC Davis vivarium, constituting the laboratory control group. Two male and two female offspring from each of seven litters were tested as follows: (1) developmental milestones at PND 4, 6, 7, 9, 10, and 12, (2) pup USV at PND 5, (3) reciprocal social interaction at PND 34–36, and (5) open field behavior at PND 42–43.

At a later timepoint, a third group of dams was employed as a control for the approximately 1.5-h vehicular transport required to move the prior groups of dams to the roadside exposure facility. Two weeks after being paired with a male breeder, all dams were singly-housed and half of the group was driven halfway to the roadside tunnel site (~45 min drive) and then back to UC Davis. The other half of the group of dams remained unmoved at the UC Davis vivarium, constituting the control group for the transported group. All of the dams and their offspring remained housed at the UC Davis vivarium for the duration of the study. Two male and two female offspring from each of 11 dams were tested as follows: (1) developmental milestones at PND 4, 6, 7, 9, 10, and 12, (2) pup USV at PND 5, and (3) open field behavior at PND 38–41.

All offspring remained in the location and exposure condition into which they were born. Behavioral testing was conducted in testing rooms adjacent to each vivarium. Two male and two female offspring from each litter were tested. To minimize carry-over effects from repeated testing, assays were performed in order from least to most stressful and at least 48 h elapsed between tests.

At separate timepoints, two additional cohorts of male and female Sprague-Dawley rats were used to collect laboratory control data for the learning and memory paradigms. These data were collected at the UC Davis vivarium prior to the testing equipment being relocated to the roadside exposure facility. Both groups remained housed at the UC Davis vivarium for the duration of the study, were well-handled prior to testing, and were offspring of Sprague-Dawley breeders who remained housed at the UC Davis vivarium. One cohort of rats was sampled from five litters and tested in the novel object recognition test at PND 45–53 and a second cohort was sampled from seven litters and tested in the fear conditioning assay at PND 42–44.

### Roadside exposure facility

Data from the CHARGE study found residential proximity to freeways to be a risk factor for NDDs when maternal residence was <309 m from a major roadway^28,44^. In order to generate toxicological data that complements the epidemiological data, the exposure facility that we employed in this study was designed to model near roadway exposures of air pollution, noise, and vibration, the same stressors experienced by people living in this environment. We drew air from the eastern face of the tunnel, not from within the tunnel itself, so that the air pollution was somewhat diluted already. Additionally, we installed vibration isolators on the feet of the exposure chambers to reduce vibration and insulated the building to reduce noise below the IACUC-mandated maximal tolerated limit of 85 decibels. Such measures were unnecessary for the UC Davis vivarium and adjacent laboratory testing rooms, which have ambient noise levels of only 64 and 43–47 decibels, respectively.

The dual housing and exposure facility, located adjacent to a major freeway tunnel system in the Bay Area of northern California, was composed of three rooms: one containing equipment for adjusting air temperature and flow, and measuring air pollutant concentrations; a second room for the two exposure chambers; and a third room for behavior testing. Each exposure chamber was 12.8 ft l × 3 ft w × 7.8 ft h and capable of accommodating 108 cages with filter tops removed. In order to minimize noise stress, all pumps and blowers were housed outside the facility and plumbed through walls. The room containing the exposure chambers and the behavioral testing suite were also additionally insulated to block noise.

Air supplied to the TRAP-exposed animals was drawn directly from the face of the tunnel. Flexible ducting carried air from the exit of the tunnel’s two eastbound bores to the exposure facility where rats were exposed to the tunnel air. Air supplied to the filtered air group was drawn from the outside of the exposure facility, where pollutant concentrations were expected to be much lower than at the tunnel face. This air was subjected to several emissions control technologies coupled together in series prior to being plumbed to the exposure chamber. These included (a) a pre-filter for removing large debris and coarse particulate matter (PM), (b) inline activated carbon filters for removing gas-phase volatile and semi-volatile organic compounds, (c) barium oxide-based catalytic converters for removing NO_x_ and (d) ultrahigh efficiency Teflon-bound glass microfiber filters for removing fine and ultrafine PM. Flow rate control and temperature conditioning were also included in compliance with IACUC specifications. Pressure within each exposure chamber was monitored constantly and blowers were programmed to maintain a small negative pressure in each chamber, with the TRAP chamber drawing in air from the tunnel and the filtered air chamber drawing in air from the outside via the filtration system.

### Behavioral testing

Two males and two females from each litter were randomly selected as behavioral test subjects and were tested on all behavioral assays with the exception of 16 animals who were only tested as pups and not as juveniles. This was to carefully control for the effect of the litter, previously described as being the most influential factor in developmental toxicological exposure studies^46,47^. Rats at the roadside exposure facility were removed from the home exposure chambers for testing and then immediately returned to the chamber following the completion of each test. For behavioral tests involving bedding, the same type of bedding as present in home cages was used.

#### Developmental milestones

Pup developmental milestones were assessed at PND 4, 6, 7, 9, 10, and 12 similarly to methods described previously^48–50^. Body length (cm; nose to tail base) and body weight (grams) were measured. Rooting reflex was measured as a turn of the head to whisker stimulation. Forelimb grasping was measured as grasping of a bar being moved upward along both front paws.

#### Isolation-induced pup ultrasonic vocalizations

During the first 2 weeks of life, rodent pups will emit ultrasonic vocalizations (USV) upon separation from their mothers and littermates^51–53^. On PND 5, isolation-induced USVs were collected from each pup for three min. A pup was randomly selected from the nest, placed in a small, open top container with bedding, and emitted USV were collected using Avisoft-RECORDER (Avisoft Bioacoustics, Glienicke, Germany) as described previously^48,54^. The container was cleaned with 70% ethanol and new clean bedding was added between each animal. USV were displayed as spectrograms and counted by a trained observer blinded to group using Avisoft-SASLab Pro (Avisoft Bioacoustics, Glienicke, Germany).

#### Juvenile reciprocal social interaction

Each rat was paired with an unfamiliar strain-, age-, and sex-matched stimulus rat and allowed to freely interact for 10 min in a clean, empty test arena (41.3 cm l × 41.3 cm w × 29 cm h) containing bedding. Behaviors were video recorded through the arena’s transparent front wall and later scored by a trained observer blinded to group as described previously^54^. Both subject and stimulus animals were isolated for 30 min prior to the test session. Subject and stimulus animals were always from different litters and stimuli rats used at the roadside facility were housed in filtered air. All behaviors scored were those of the subject animal. Behaviors scored for duration were: (1) exploring, (2) following or chasing, (3) social sniffing, (4) anogenital sniffing, and (6) self-grooming. The testing room was illuminated to ~30 lux.

#### Open field exploration

In order to control for the potentially confounding effects of hypo- or hyperactivity on the other behavioral assays, exploratory activity in a novel open arena was evaluated over a 30 min session. Rats were placed in the center of the arena at the start of the testing session. Using methods similar to those previously described^48,54^, total distance traveled and time spent in the center of the arena were measured using one of two comparable automated systems: an opaque matte black arena (54.1 cm l × 54.1 cm w × 34.3 cm h) equipped with video tracking software (EthoVision XT 12; Noldus Information Technology, Wageningen, Netherlands) or the fully automated Digiscan Animal Activity Monitors with Integra software (Omnitech Electronics, Columbus, OH, USA). The testing room was illuminated to ~30 lux.

#### Novel object recognition

Novel object recognition was assayed using methods similar to those described previously^49,55^. Using an opaque matte black box (54.1 cm l × 54.1 cm w × 34.3 cm h), each animal was habituated to the empty arena for 30 min on the day prior to the test. On the day of the test, each subject was again habituated to the arena for 30 min before two identical objects were placed gently in the arena with the animal. After a 10 min familiarization session, the animal was isolated in a clean holding cage with bedding for 60 min. During this time, the arena and the objects were cleaned with 70% ethanol and one clean familiar object and one clean novel object were placed in the original positions of the two identical objects during familiarization. Both the identity and location of the novel object within the arena were counterbalanced to address potential inherent object preferences or side biases. Our protocol has been published as standard by the Intellectual and Developmental Disability’s Behavior Cores^56^. Upon being returned to the arena for the recognition test, the subject was allowed 5 min to interact with the familiar and novel objects. Time spent sniffing each object during each phase of testing was automatically measured via video tracking software (EthoVision XT 10 and 12; Noldus Information Technology, Wageningen, Netherlands). Objects used were orange plastic cones (8.5 cm l × 8.5 cm w × 9.5 cm h) and glass bell jars (7.5 cm d × 10.3 cm h). The testing room was illuminated to ~30 lux.

#### Contextual and cued fear conditioning

Contextual and cued fear conditioning was carried out using an automated fear conditioning chamber (Med Associates, Inc., Fairfax, VT, USA) similar to methods described previously^49,57^. During training on day one, rats were exposed to a series of three noise-shock (CS-US) pairings in a testing chamber with specific visual, odor, and tactile cues. The training environment was brightly lit (~100 lux), contained a metal wire floor, and included 0.3 mL of vanilla odor cue (1:100 dilution of McCormick Vanilla Extract). White noise (80 dB) was played for 30 s and a foot shock (0.7 mA) occurred during the final two sec of the noise cue. A two min period for exploration preceded the first noise-shock pairing and elapsed between each noise-shock pairing. A 30 s exploration period followed the final noise-shock pairing and the entire training session was eight min in duration. On day two of testing, the subject was placed back inside the training environment for five min. The chamber contained identical contextual cues as the training session, but no white noise or foot shock occurred. On day three of testing, the subject was placed back inside the training environment for 6 min, but the chamber context was altered. The overhead lighting was turned off and the chamber contained a novel smooth plastic floor, novel black angled walls, and a novel lemon scent (1:100 dilution of McCormick Lemon Extract). An initial three min exploration period was followed by a three min presentation of the white noise conditioned stimulus. Time spent freezing during each test phase was automatically measured by the VideoFreeze software (version 2.7; Med Associates).

### Statistical analysis

Particulate matter concentrations were compared using paired *t*-test since measurements occurred on the same days in both groups. Vocalizations were analyzed via unpaired (Student’s) *t*-test for two groups or via one-way ANOVA with Tukey’s multiple comparisons post hoc test for three groups. Developmental metrics and open field parameters were analyzed via two-way repeated measures ANOVA with exposure as the between-group factor and time as the within-group factor. Significant ANOVAs were followed by Tukey’s post hoc testing. Log-Rank (Mantel-Cox) test was used to compare the percentage of animals achieving developmental milestones. Social interaction parameters were compared with one-way ANOVA followed by Tukey’s post hoc testing. Comparisons between sniff times of objects were made within each exposure group via paired *t*-test and comparisons between freezing times were compared within test day with repeated measures ANOVA (for training and cued freezing) or unpaired (Student’s) *t*-test (for contextual freezing). Group sizes were chosen based on past experience and power analyses^58^, and data were analyzed with GraphPad Prism. Behavioral data passed distribution normality tests, were collected using continuous variables, and thus were analyzed via parametric tests. Variances were similar between groups and data points within two standard deviations from the mean were included in analyses. All significance levels were set at *p* < 0.05 and all *t*-tests were two-tailed. Multiple comparisons were corrected for via post hoc testing via Tukey’s multiple comparisons test. Data are presented as mean ± standard error of the mean.

## Results

### Reproductive success

**Fig. 1:**
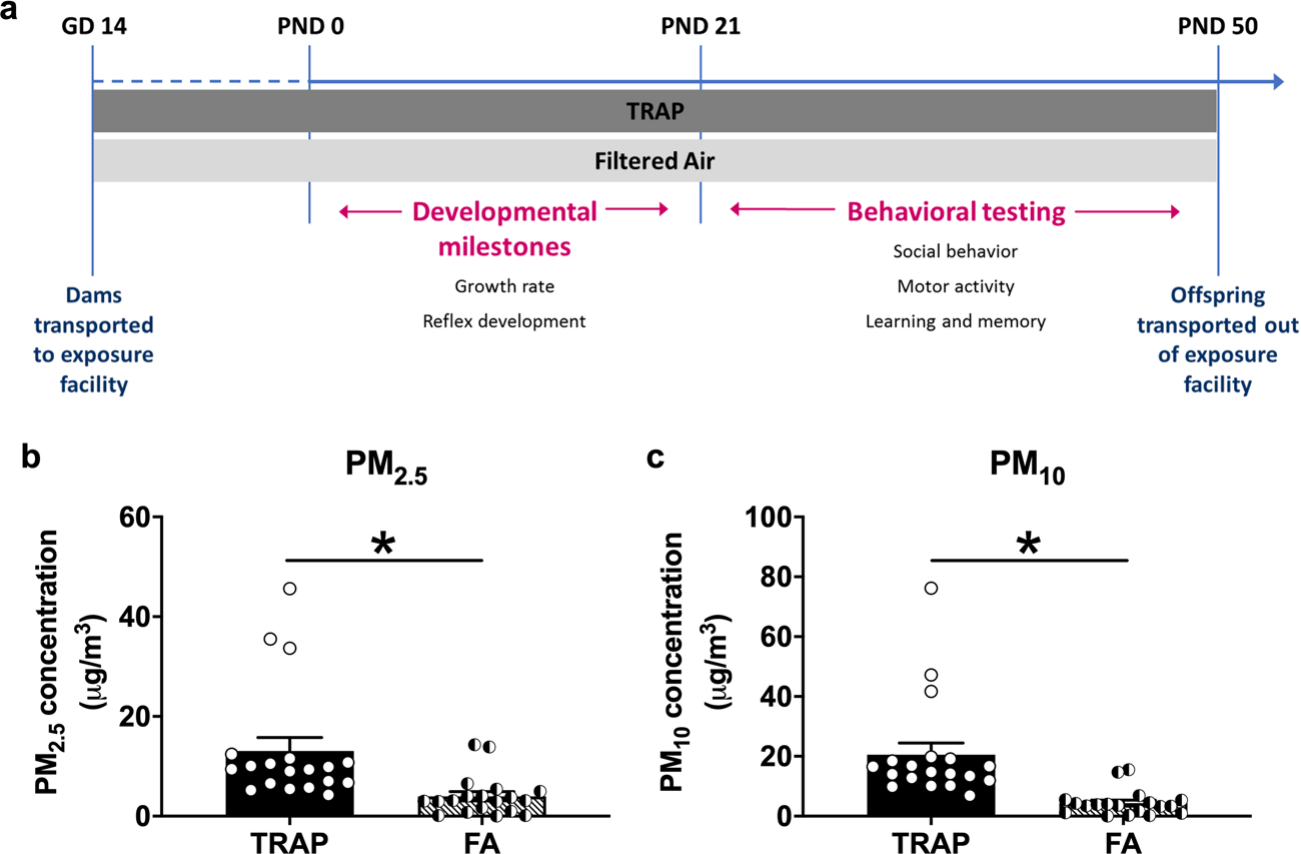
Timeline and quantification of roadside TRAP exposure. **a** Pregnant female rats were transported to the roadside exposure facility at approximately gestational day (GD) 14 and were randomly assigned to be housed in either the TRAP or filtered air (FA) exposure chambers. Offspring, remained in the exposure condition into which they were born, were tested on a variety of developmental milestone assays between four days after birth (postnatal day (PND) 4) and weaning at PND 21 and then a battery of standardized behavioral assays between PND 21 and 50. **b**, **c** Particulate matter (PM) concentrations of the TRAP air and FA were quantified on 19 days. Both **b** PM_2.5_ and **c** PM_10_ concentrations of the TRAP air were significantly higher than those of the FA. *p < 0.05, paired *t*-test.

Two of three groups of pregnant female rats were transported to the roadside exposure facility at approximately gestational day (GD) 14, while the third group remained in the laboratory at UC Davis. Dams of the roadside cohort were randomly assigned to be housed in either the TRAP or filtered air (FA) exposure chamber. In the laboratory control setting, 10 of 11 dams gave birth. One litter was cannibalized and did not survive to PND 2. We assayed a final litter count of 9. In the FA-exposed group at the roadside facility, 17 of 18 dams gave birth. One litter was cannibalized and did not survive to PND 2, so we assayed a final litter count of 16. In the TRAP-exposed group at the roadside facility, 10 of 10 dams gave birth. The average number of days between arrival at the roadside vivarium and birth was 10 days for both exposure groups, and there was no effect of group on litter size nor male to female ratio (Supplementary Table S1). Figure 1a illustrates our experimental design described in the methods.

### Particulate concentrations in TRAP and FA exposures

Twenty-four-hour PM_2.5_ and total suspended particulate mass concentrations measured immediately upstream of the FA and TRAP exposure chambers at the roadside tunnel facility for the study duration are described with extensive detail in Fig. S1 and Bein et al. (under review)^43^. A unique and defining characteristic of our design is that it captured significant diurnal and day-to-day variations in exposure concentrations that cannot be readily recreated in the laboratory. These variations were easily seen in the size distribution of particle number concentrations (Fig. S1) and described comprehensively in Bein et al. (under review)^43^. Figure 1b, c illustrate the clearly increased PM_2.5_ and PM_10_, respectively, in the tunnel-sampled air (TRAP) compared to filtered air (FA), thereby validating our exposure system (Fig. 1b*t*_(1, 18)_ = 4.562, *p* < 0.001 and Fig. 1c*t*_(1, 18)_ = 4.923, *p* < 0.001).

### Reduced isolation-induced pup ultrasonic vocalizations (USV)

**Fig. 2:**
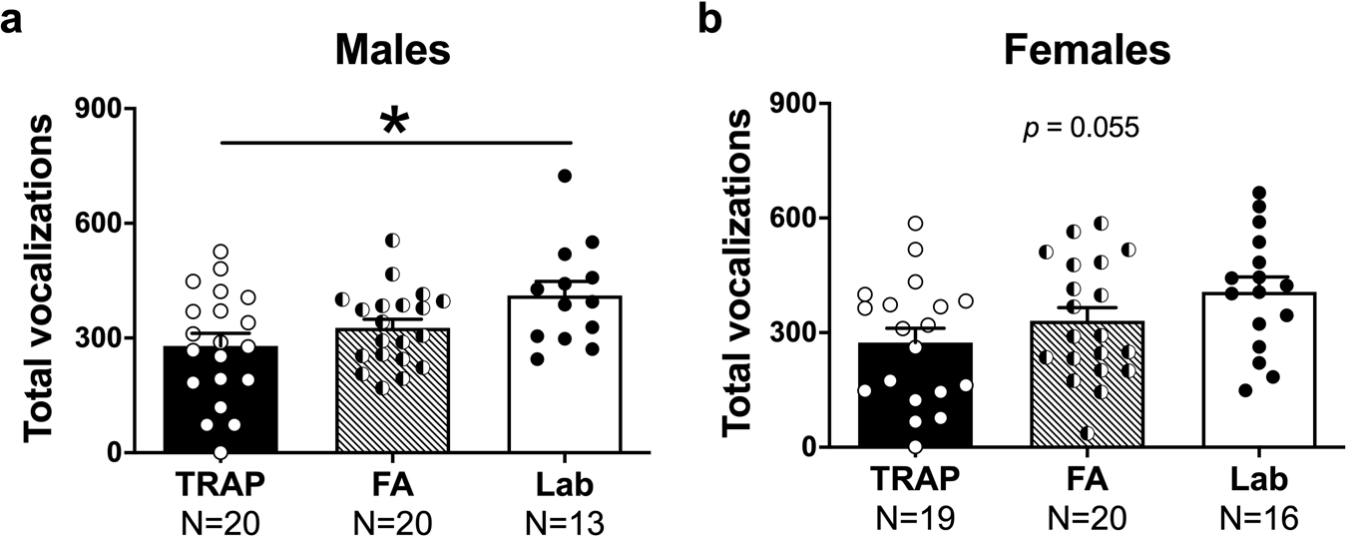
Reduced isolation-induced ultrasonic vocalizations (USV) of TRAP-exposed pups at PND 5. **a** Male pups exposed to TRAP emitted significantly fewer USV during the three min isolation compared to lab controls. **b** Exposure did not affect USV emission in females, although the trend indicated reduced numbers of calls in the TRAP group compared to lab controls. *p < 0.05, one-way ANOVA followed by Tukey’s multiple comparisons test.

Isolation-induced USV were collected for 3 min as social communication signals in rat pups on PND 5, as previously described^48,54^. In male offspring, a significant effect of exposure on USV was discovered (Fig. 2a*F*_(2, 50)_ = 4.287, *p* < 0.02). TRAP-exposed pups emitted the fewest USV calls (*p* = 0.014 versus laboratory controls) and, interestingly, the FA-exposed group also trended to emit lower calls compared to the laboratory control group (*p* = 0.154). Raw values show the phenomenon that TRAP had the lowest number of calls: non-significant but noteworthy effects on USV by exposure group using mean ± SD showed that in male laboratory controls USV were 412 ± 132.8, FA USV were 327 ± 99.90, and TRAP USV were 280 ± 145.3. Given that TRAP-exposed did not differ from FA-exposed by Tukey’s multiple comparisons post hoc analysis (*p* = 0.475), we cannot conclude that the air quality alone caused the lower numbers of USV. Although, the SD of the raw values allows us to see the high variability in call numbers by group.

A similar pattern was illustrated in the female offspring (Fig. 2b*F*_(2, 52)_ = 3.069, *p* = 0.055) albeit statistical significance in the overall ANOVA was not <0.05. Non-significant but noteworthy effects on USV by exposure group using mean ± SD showed that in female laboratory controls USV were 407 ± 154.60, FA USV were 331 ± 155.7, and TRAP USV were 275 ± 162.20. However, as the overall ANOVA was not under *p* = 0.05, we did not run post hoc analyses. Given the effect of the roadside exposure (TRAP and FA) in males and trend in females, we were unable to extract a sound statistical finding on calls that resulted from our intermittent, intensity varying, mixture of real-world pollution in the TRAP group. Trends, raw values, and high SD allow us to see the high variability in call numbers by group.

Body weight and temperature were also collected since body temperature is known to alter pup USV emission^51,59–62^. Weights and temperature did not differ by roadside air exposure (weight TRAP versus FA *t*_(1, 38)_ = 0.753, *ns* and temperature TRAP versus FA *t*_(1, 38)_ = 1.375, *ns*). On PND 5, males of both roadside exposed groups weighed less than laboratory controls (TRAP versus lab *t*_(1, 31)_ = 2.603, *p* < 0.02; FA versus lab *t*_(1, 31)_ = 2.388, *p* < 0.03).

### Delayed growth and milestone achievement of both TRAP and FA-exposed pups

**Fig. 3:**
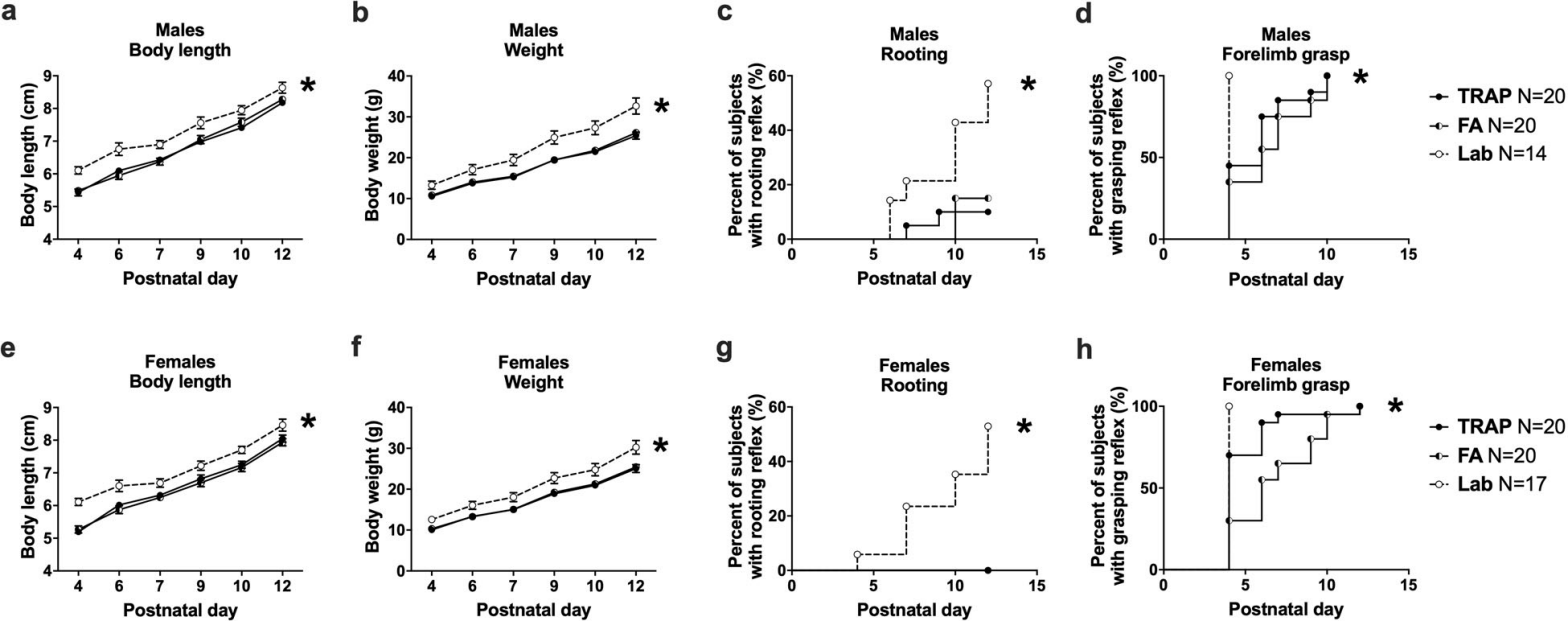
Delayed growth and milestone achievement of roadside TRAP- and FA-exposed pups. **a** Male pups exposed to TRAP or FA had significantly reduced body length and **b** body weight throughout early development compared to lab controls. Males of both roadside groups exhibited a significant delay in the development of **c** rooting and **d** forelimb grasping reflexes. **e** Female pups exposed to TRAP or FA also had reduced body length and **f** body weight and developed **g** rooting and **h** forelimb grasping reflexes later than lab controls. **a**, **b**, **e**, **f** *p < 0.05, repeated measures ANOVA followed by Tukey’s multiple comparisons test. **c**, **d**, **g**, **h** *p < 0.05, Log-Rank (Mantel-Cox) test.

Figure 3a–h shows delayed early physical development and neurological reflexes in TRAP- and FA-exposed offspring compared to laboratory controls. All male and female subjects gained weight and grew in length over time (males Fig. 3a_length_ *F*_(5, 255)_ = 390.8, *p* < 0.0001; Fig. 3b_weight_ *F*_(5, 255)_ = 1186, *p* < 0.001 and females Fig. 3e_length_ *F*_(5, 270)_ = 322.1, *p* < 0.0001; Fig. 3f_weight_ *F*_(5, 255)_ = 1092, *p* < 0.001). Significant effects of exposure on body length and weight were discovered in both sexes (males Fig. 3a_length_ *F*_(2, 51)_ = 12.66, *p* < 0.001; Fig. 3b_weight_ *F*_(2, 51)_ = 10.04, *p* < 0.001 and females Fig. 3e_length_ *F*_(2, 54)_ = 13.05, *p* < 0.001; Fig. 3f_weight_ *F*_(2, 54)_ = 6.312, *p* < 0.004). TRAP-exposed (males *p* < 0.001 and females *p* < 0.006) and FA-exposed (males *p* < 0.001 and females *p* < 0.02) offspring differed from laboratory controls in both length and weight. Interestingly, no differences were observed between TRAP- and FA-exposure for length (males *ns* and females *ns*) or weight (males *ns* and females *ns*). The rooting and grasping reflexes were delayed in both the TRAP- and FA-exposed offspring compared to laboratory controls in both males (TRAP Fig. 3c_rooting_ Log-rank *χ*^2^_(1)_ = 8.35, *p* < 0.005; FA Fig. 3c_rooting_ Log-rank *χ*^2^_(1)_ = 7.18, *p* < 0.01; TRAP Fig. 3d_grasping_ Log-rank *χ*^2^_(1)_ *=* 11.05, *p* < 0.001; FA Fig. 3d_grasping_ Log-rank *χ*^2^_(1)_ *=* 14.30, *p* < 0.001) and females (TRAP Fig. 3g_rooting_ Log-rank *χ*^2^_(1)_ *=* 13.98, *p* < 0.001; FA Fig. 3g_rooting_ Log-rank *χ*^2^_(1)_ *=* 13.98, *p* < 0.001; TRAP Fig. 3h_grasping_ Log-rank *χ*^2^_(1)_ *=* 5.92, *p* < 0.05; FA Fig. 3h_grasping_ Log-rank *χ*^2^_(1)_ *=* 18.63, *p* < 0.001). Additional developmental milestones are shown in Supplementary Table S2.

### Juvenile reciprocal dyad social interactions (social play)

**Fig. 4:**
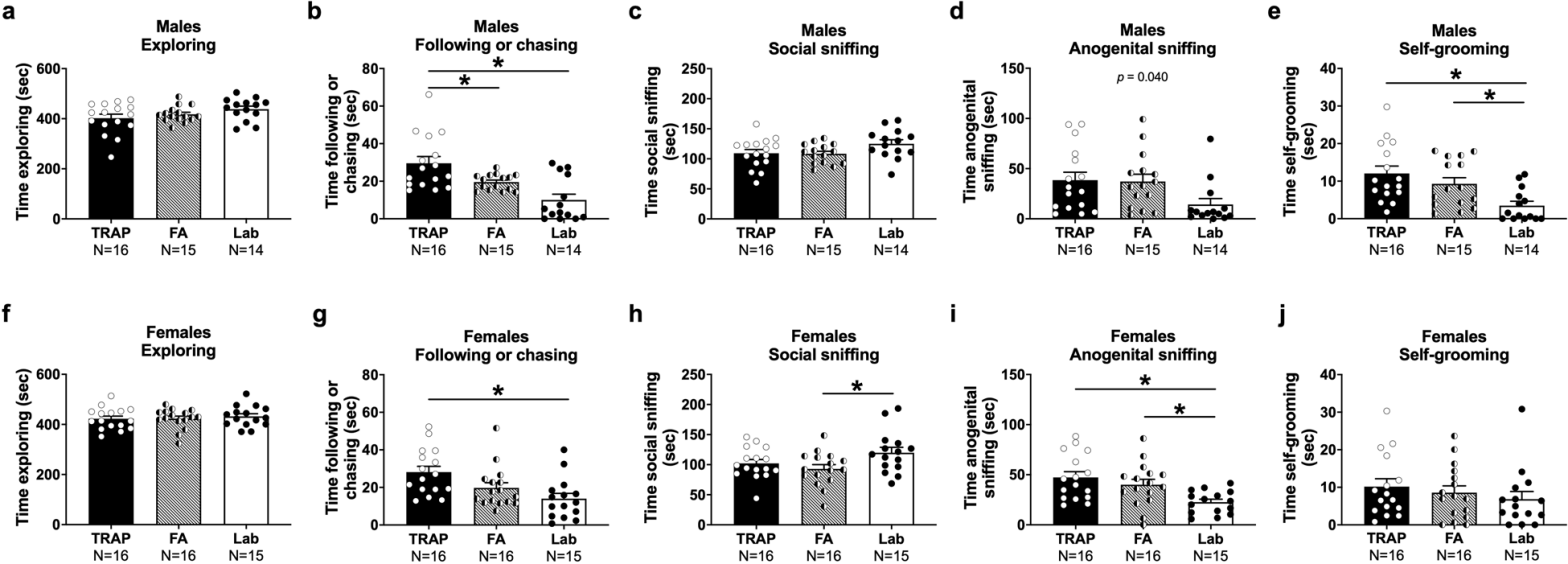
Roadside TRAP- and FA-exposed rats differed from lab controls during juvenile reciprocal social interactions on several key parameters. **a** Roadside exposures did not affect levels of exploration during the social interaction assay for males, however **b** TRAP-exposed males spent significantly more time following or chasing the stimulus animal than did FA-exposed or lab controls. **c** Roadside-reared males showed typical levels of social sniffing but **d** there was a significant effect of group on anogenital sniffing, with post hoc trends suggesting that both roadside exposure groups spent more time anogenital sniffing compared to lab controls. **e** Both TRAP- and FA-exposed males spent more time self-grooming than lab controls. **f** Females of all groups exhibited comparable levels of exploration, but **g** TRAP-exposed females spent more time following or chasing than lab controls. **h** FA-exposed females spent significantly less time social sniffing relative to lab controls and **i** both roadside groups had elevated levels of anogenital sniffing. **j** Females of all groups displayed similar levels of self-grooming. *p < 0.05, one-way ANOVA followed by Tukey’s multiple comparisons test.

Male and female subject exploration did not differ between exposure groups and neither exposure group differed from laboratory controls (males Fig. 4a*F*_(2, 42)_ = 2.065, *ns* and females Fig. 4f*F*_(2, 44)_ = 0.2467, *ns*). This key information suggests that any differences in social behavior are not confounded by motor abilities, or hypo-, or hyper-exploration of the arena. Levels of this parameter were comparable and consistent with earlier findings using Sprague-Dawley rats at this age^63–68^ and with our transported laboratory-tested control group (Fig. S2).

Social deficits, by an unusually high amount of time on the play parameter of following/chasing, were observed in both sexes (males Fig. 4b*F*_(2, 42)_ = 11.61, *p* < 0.001 and females Fig. 4f*F*_(2, 44)_ = 5.944, *p* < 0.006). Specifically, TRAP-exposed males spent more time following/chasing compared to FA-exposed males (*p* < 0.05) and laboratory controls (*p* < 0.001). FA-exposed males also trended to spend more time following/chasing compared to laboratory controls (*p* = 0.06). TRAP-exposed females exhibited a strong trend to spend more time following/chasing compared to FA-exposed females (*p* = 0.10) and TRAP-exposed females spent more time following/chasing compared to laboratory controls (*p* < 0.004). FA-exposed females did not differ on time spent following/chasing compared to laboratory controls (*p* = 0.356).

In females, exposure had a significant effect on time engaged in the key interaction metric of social sniffing, which includes nose-to-nose sniffing, neck and body sniffing, and other bouts of contact sniffing with the partner stimulus rat (females Fig. 4g*F*_(2, 44)_ = 3.264, *p* < 0.05). The TRAP and FA-exposed groups did not differ from one another (*p* = 0.650). Interestingly, FA-exposed (*p* = 0.040) females spent less time engaged in social sniffing compared to laboratory controls but the TRAP-exposed female group did not differ from laboratory controls (*ns*). In contrast, only a trending difference between groups was observed in the key metric of social sniffing in males (males Fig. 4c*F*_(2, 42)_ = 2.622, *p* = 0.085).

Nose-to-anogenital sniffing time, when initiated by the subject rat, was significantly affected in both sexes (males Fig. 4d*F*_(2, 42)_ = 3.492, *p* = 0.040 and females Fig. 4i*F*_(2, 44)_ = 5.944, *p* < 0.006). TRAP and FA-exposed groups did not differ from one another (*ns*). Although neither the FA-exposed males (*p* = 0.079) nor the TRAP exposed males (*p* = 0.056) significantly differed compared to laboratory controls in anogenital sniffing upon post hoc analyses, trending differences were discovered. Whereas this parameter did not differ in the transport control group (Fig. S2) suggesting the cause was the roadside exposure conditions and not the transport during gestation.

Time spent engaged in self-grooming differed between exposure groups in males (Fig. 4e*F*_(2, 42)_ = 6.870, *p* < 0.004) but not females (Fig. 4j*F*_(2, 44)_ = 0.6994, *ns*). Tukey’s multiple comparisons post hoc analysis revealed that both the TRAP (*p* = 0.049) and FA-exposed (*p* = 0.002) male groups exhibited higher self-grooming scores compared to laboratory controls. Social interaction metrics that did not differ between the transported group and laboratory control offspring are illustrated in Fig. S2 and additional play metrics that did not differ between groups are summarized in Supplementary Table S3.

### Normal exploratory locomotor behavior in an open field arena

**Fig. 5:**
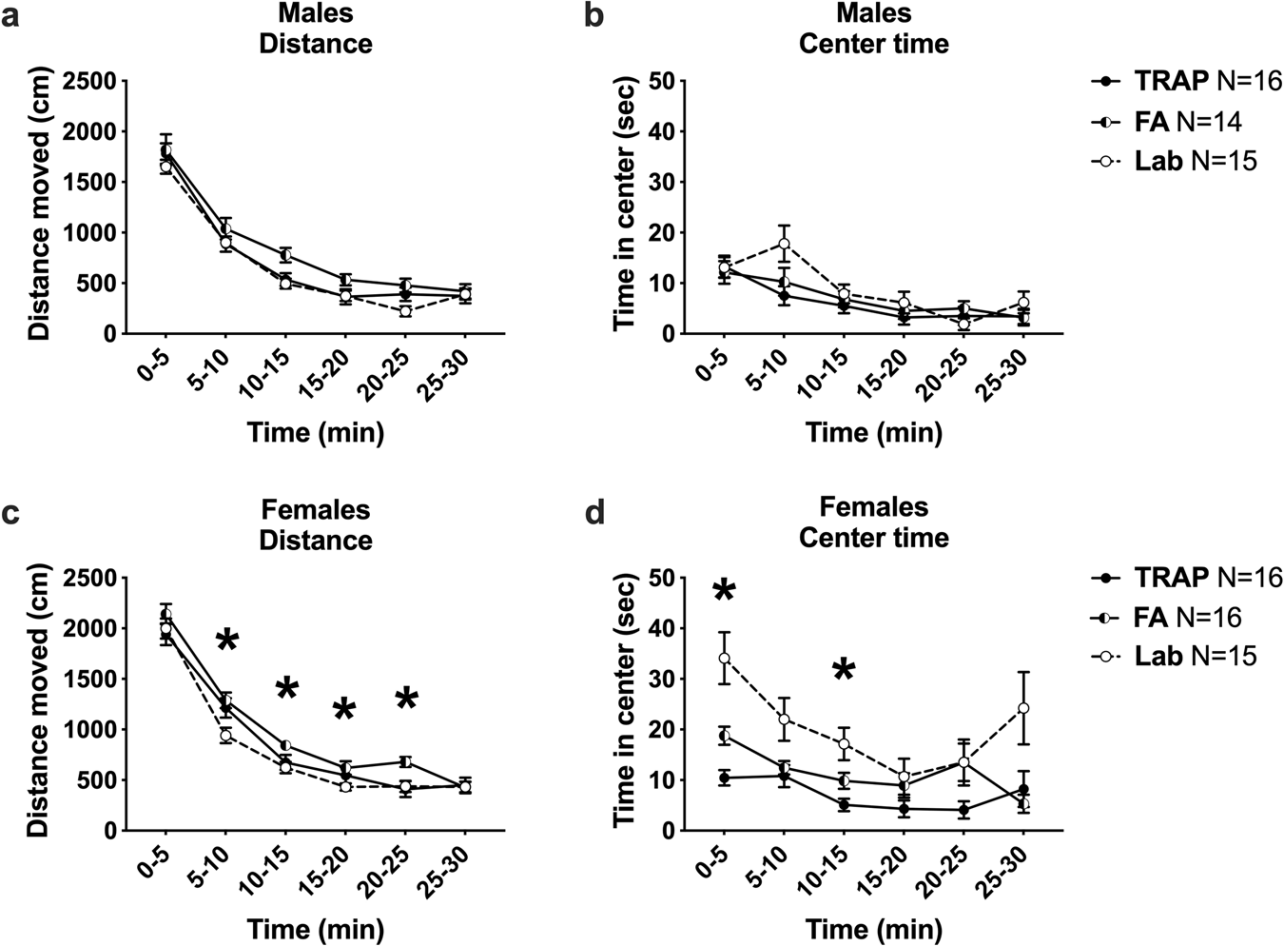
Atypical exploratory activity in a novel open field in rats exposed to roadside TRAP and FA. **a** Roadside exposures did not affect males’ gross locomotion or **b** time spent in the center during a 30-min exploration of a novel arena. **c** In females, there was a significant effect of group on distance moved, with trends suggesting that FA-exposed females covered more distance during the assay compared to lab controls. **d** TRAP-exposed females spent less time in the center than did FA-exposed females, and both TRAP- and FA-exposed females displayed significantly reduced center time relative to lab controls. *p < 0.05, repeated measures ANOVA followed by Tukey’s multiple comparisons test.

Motor abilities were tested in an open field assay, assessing cm of distance traveled using beam breaks and time spent in the center of the arena. FA- and TRAP-exposed juvenile male rats, as well as a cohort of male laboratory controls, exhibited no group differences in total activity (Fig. 5a*F*_(2, 42)_ = 3.042, *ns*). As expected, all groups decreased activity over time (Fig. 5a*F*_(4, 151)_ = 220.2, *p* < 0.0001). No treatment group differences were detected in center time measures in males (Fig. 5b*F*_(2, 42)_ = 1.367, *ns*). Group effects were observed in FA- and TRAP-exposed juvenile female rats, as well as a cohort of female laboratory controls, in total activity (Fig. 5c*F*_(2, 44)_ = 4.690, *p* < 0.02). There was not a significant difference in performance between TRAP- and FA-exposed rats, except at a single timepoint (20–25 min: *p* = 0.019). TRAP did not differ from the laboratory controls (*ns*) at any timepoint, while the FA-exposed group and lab controls differed at four timepoints upon post hoc analyses in females (5–10 min: *p* = 0.009; 10–15 min: *p* = 0.011; 15–20 min: *p* = 0.049; 20–25 min: *p* = 0.009). Group differences were detected in center time measures in females (Fig. 5d*F*_(2, 44)_ = 10.39, *p* < 0.001). As expected, all groups decreased center time across the 30-min testing session (Fig. 5a*F*_(3, 140)_ = 8.70, *p* < 0.0001). TRAP-exposed females exhibited lower time in the center compared to FA-exposed rats (*p* = 0.007). Both TRAP (0–5 min: *p* = 0.001; 10–15 min: *p* = 0.007) and FA-exposed (0–5 min: *p* = 0.030) females groups differed by lower center times compared to the laboratory controls upon post hoc analyses.

### Intact object recognition and Pavlovian conditioning learning and memory behavior

**Fig. 6:**
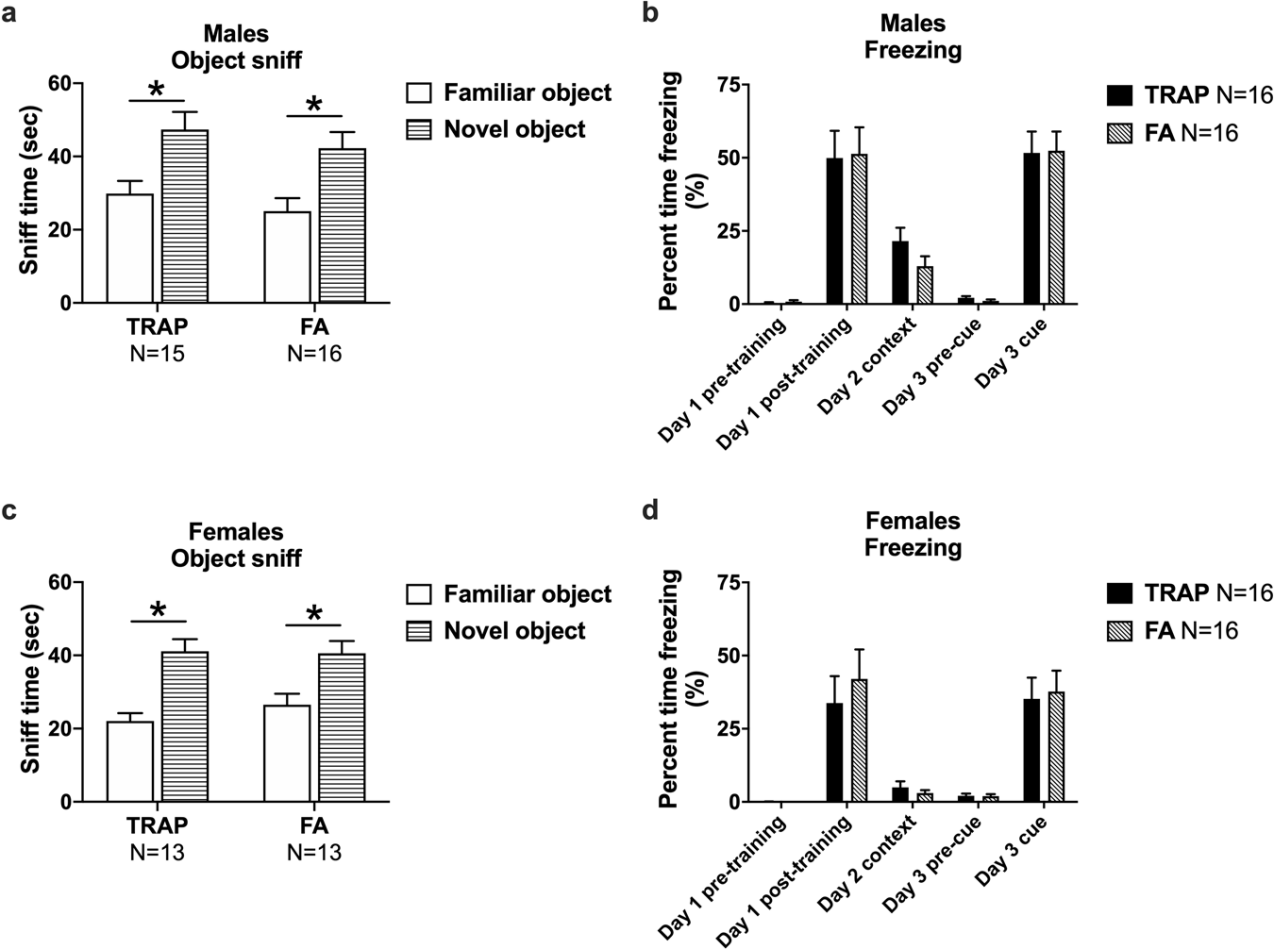
Learning and memory in roadside exposed rats. **a** Males exposed to TRAP or FA displayed intact novel object recognition as evidenced by spending significantly more time sniffing the novel object than the familiar object. **b** Exposure to TRAP did not affect contextual or cued fear memory in males and both TRAP and FA groups displayed high levels freezing day 1 post-training and to the cue presentation on day 3. **c** Both groups of roadside exposed females spent significantly more time investigating the novel object compared to the familiar object and **d** no group differences were observed in percent time freezing during the test of contextual and cued memory. **a**, **c** *p < 0.05, paired *t*-test, familiar vs. novel. **b**, **d** *p < 0.05, Day 1, 3: repeated measures ANOVA; Day 2: Student’s *t*-test, TRAP vs. FA.

Manual and automated scoring indicated both male TRAP- and FA-exposure groups spent more time investigating the novel object versus the familiar object, thereby exhibiting typical novel object preference (Fig. 6a TRAP-exposed, *t*_(1, 15)_ = 3.269, *p* < 0.006 and FA-exposed, *t*_(1, 15)_ = 3.081, *p* < 0.008). Times spent exploring the objects during the familiarization component were similar for both groups using mean ± SEM in that FA-exposed sniffing investigation times were 135.5 ± 15.2 s, and TRAP-exposed sniffing investigation times were 110.3 ± 12.1 s. Similarly, both female exposure groups spent more time investigating the novel object versus the familiar object, exhibiting typical novel object preference (Fig. 6c TRAP-exposed, *t*_(1, 12)_ = 4.316, *p* < 0.001 and FA-exposed, (*t*_(1, 12)_ = 3.720, *p* < 0.003). Thus, roadway exposure did not adversely affect object learning or short-term memory recall. This negative finding was not the result of a lack of participation or object investigation as times spent exploring the objects during the familiar exposure component, in females, were similar for both groups using mean ± SEM in that FA-exposed sniffing investigation times were 138.6 ± 6.8 s, and TRAP exposed sniffing investigation times were 117.0 ± 9.4 s. Object sniff times observed 60 min following familiarization with one object type in laboratory control subjects (males Fig. S3a*t*_(1, 15)_ = 3.997, *p* = 0.001 and females Fig. S3b*t*_(1, 14)_ = 2.788, *p* = 0.015) illustrated typical novel object preference in groups run in our rat behavioral core when given the opportunity to investigate a novel and a familiar object.

Learning and memory were further evaluated using two measures of Pavlovian fear conditioning with a 24-h contextual component and a 48-h auditory cued fear conditioning. Significant main effects of time (males Fig. 6b *F*_(1, 30)_ = 61.06, *p* < 0.0001 and females Fig. 6d *F*_(1, 30)_ = 31.27, *p* < 0.0001) but not exposure group (males Fig. 6b *F*_(1, 30)_ = 0.0213, *ns* and females Fig. 6d *F*_(1, 30)_ = 0.3597, *ns*) or interaction (males Fig. 6b *F*_(1, 30)_ = 0.0061, *ns* and females Fig. 6d *F*_(1, 30)_ = 0.3785, *ns*) indicated that high levels of freezing were observed in both groups subsequent to the conditioned stimulus (CS)—unconditioned stimulus (UCS) pairings on the training day. Elevated post-training freezing in both exposure groups with no group difference in training freeze scores in males or females indicates no confounds and no deficits in the learning of the associations between the context stimuli and auditory cues. No difference in freezing scores was observed 24 h following CS-UCS training between TRAP- and FA-exposed subjects freezing scores in males (Fig. 6b*t*_(1, 30)_ = 1.510, *ns*) or females (Fig. 6d*t*_(1, 30)_ = 0.8535, *ns*) when placed in the context chamber from conditioning training with identical stimulus cues. Levels of freezing, between the pre- and post-cue presentation 48 h after training, revealed significant main effects of cue presentation (males Fig. 6b *F*_(1, 30)_ = 112.1, *p* < 0.0001 and females Fig. 6d *F*_(1, 30)_ = 47.86, *p* < 0.0001) but not exposure group (males Fig. 6b *F*_(1, 30)_ = 0.0006, *ns* and females Fig. 6d *F*_(1, 30)_ = 0.0484, *ns*) or interaction (males Fig. 6b *F*_(1, 30)_ = 0.0370, *ns* and females Fig. 6d *F*_(1, 30)_ = 0.0694, *ns*). Therefore, no group difference was found in freezing in response to the auditory cue between TRAP- and FA-exposed subjects when placed in the novel chamber with unique contextual cues (olfactory, visual, and textural). Freezing scores in laboratory control subjects observed 24 h following CS-UCS training compared to pre-training scores (males Fig. S4a*t*_(1, 25)_ = 5.722, *p* < 0.0001 and females Fig. S4b*t*_(1, 22)_ = 4.486, *p* < 0.001) illustrated typical fear responses in groups run in our rat behavioral core when placed in the context chamber from conditioning training with identical stimulus cues.

### Transport stress does not cause the observed behavioral phenotypes

**Fig. 7:**
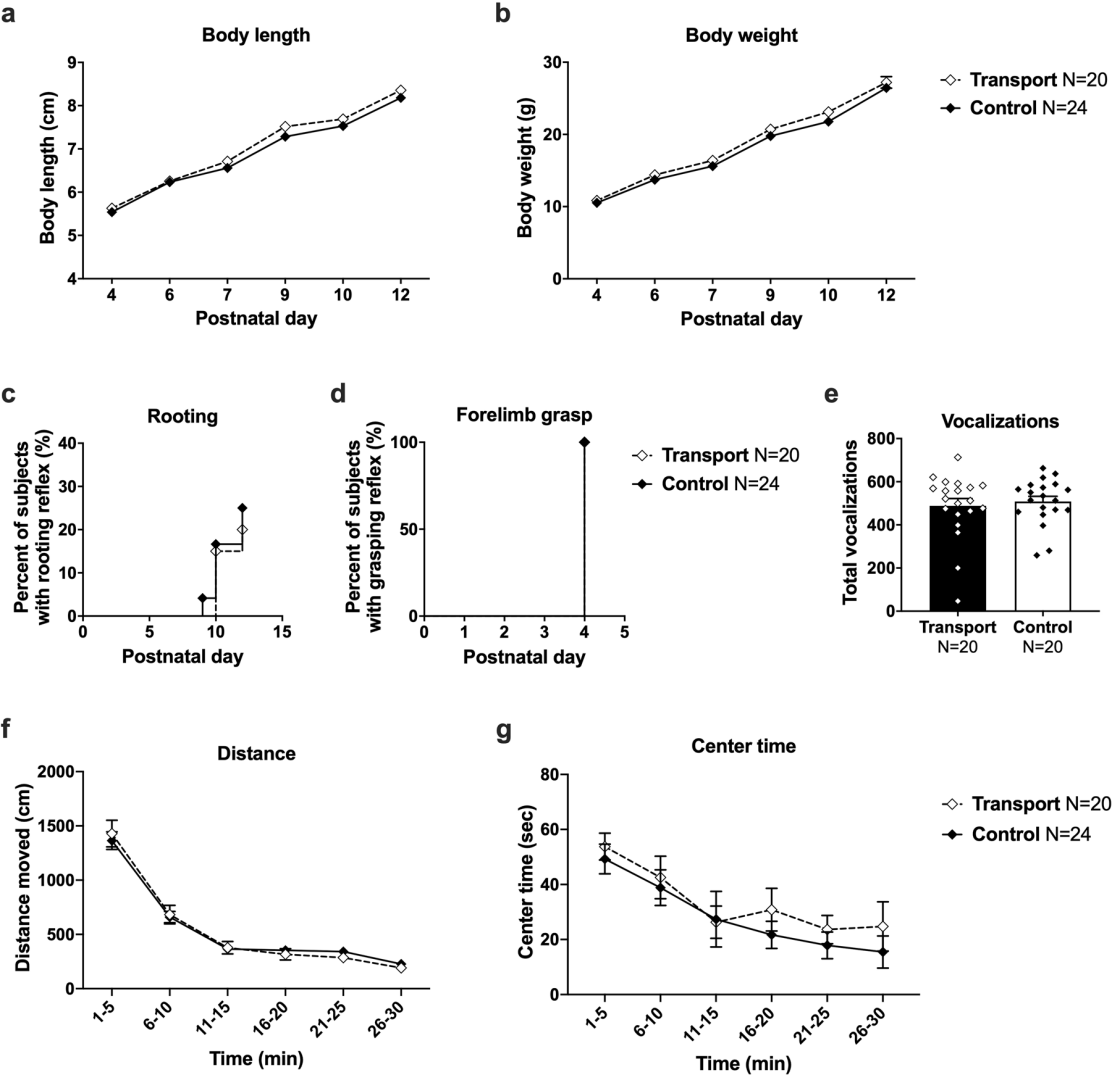
No effect of gestational transport alone on offspring development and behavior. Pups born to dams that experienced a transport event at approximately GD 14 exhibited no physical or behavioral abnormalities. **a** Body length and **b** body weight were typical throughout early life, as was the timing of the development of **c** rooting and **d** forelimb grasping reflexes. **e** Gestational transport did not affect the number of isolation-induced pup ultrasonic vocalizations at PND 5 and **f** juveniles exhibited similar exploratory activity in a novel open field as indicated by total distance moved and **g** time spent in the center. **a**, **b**, **f**, **g** *p < 0.05, repeated measures ANOVA. **c**, **d** *p < 0.05, Log-Rank (Mantel-Cox) test. **e** *p < 0.05, Student’s *t*-test.

The effect of potential stress on the pregnant dam during the transport to the roadside exposure facility was ruled out as a causal mechanism for these physical and behavioral changes. Figure 7a–d show no delay in early physical development and neurological reflexes. This figure combines sexes as no sex difference was observed throughout the developmental outcomes (Fig. 2). In transported and control offspring, all male and female subjects gained weight and grew in length over time (Fig. 7a_length_ *F*_(5, 173)_ = 846.2, *p* < 0.001; Fig. 7b_weight_ *F*_(5, 255)_ = 1186, *p* < 0.001). There was a trend but no statistically significant effect of transport on body length (Fig. 7a*F*_(1, 42)_ = 3.278, *p* = 0.077) or body weight (Fig. 7b*F*_(1, 42)_ = 1.764, *ns*). Neurological reflexes, including the rooting and grasping reflexes, were normal in transported and control offspring (Fig. 7c_rooting_ Log-rank *χ*^2^_(1)_ = 0.1716, *ns*; Fig. 7d_grasping_ Log-rank *χ*^2^_(1)_ *=* 00.00, *ns*). Figure 7e shows that there were no differences in PND 5 pup USV emissions across the transported and control offspring (Fig. 7e*t*_(1, 38)_ = 0.4814, *ns*). No differences in total activity (Fig. 7f*F*_(1, 42)_ = 0.0049, *ns*) or time spent in the center of the open field arena (Fig. 7g*F*_(1, 42)_ = 0.4879, *ns*) were observed between the transported offspring compared to control group. Overall, no effect of transport alone was observed on offspring development. Pups born to dams that experienced a transport at approximately GD 14 exhibited no physical or behavioral abnormalities (Fig. 7) and exhibited no differences in social investigative events such as exploring, social sniffing, anogenital sniffing, following/chasing, or the repetitive behavior of self-grooming (Fig. S2). Pups born to dams that experienced a transport at approximately GD 14 also exhibited no differences in social play point events such as pouncing, pinning, and pushing under or crawling over (Table S3).

## Discussion

Our goal was to corroborate human studies that have linked increased risk of NDDs to near roadway TRAP exposures. To do this, we developed an innovative exposure model that quantifies and delivers TRAP collected from a traffic tunnel to rats during both in utero and post-natal development. This design avoided limitations of single exposure paradigms, including requiring anesthesia and difficulties mimicking real-world mixtures of TRAP, while simultaneously leveraging earlier literature to yield consensus. Both roadside exposure groups had significantly delayed growth and development of psychomotor reflexes, displayed altered social interactions, and exhibited abnormal activity in an open field compared to lab controls. This is the first report that used carefully controlled subgroups to illustrate that developmental exposure to realistic near roadway exposures caused subtle but significant changes in developmental endpoints and functional outcomes (i.e., behavior). This confirms the theory suggested by epidemiological studies that in addition to TRAP, noise, vibration, and proximity to highways may be additional risk factors for NDDs in combination with genetic susceptibility or independently^19,20,28,69–73^. Our work presented herein is also a novel, important addition because, to our knowledge, this is the first nonclinical study that did not use high levels of particulate matter (PM), concentrated ambient ultrafine particles (CAPS), and/or diesel exhaust and discovered subtle but reportable behavioral outcomes. These findings support the need for further research delineating causal link(s) between exposure to TRAP and behavioral outcomes relevant to NDDs and adding to our understanding of the risks posed by air pollution to the developing nervous system.

Recently, a few well recognized laboratories have used reductionist experimental designs to investigate the effects of diesel exhaust^69,70,74,75^ and particulate matter (sizeable and ultrafine)^38,76–81^, the components most implicated in mediating the neurotoxic effects of TRAP. We extended this published research with our innovative real-world exposure to a dynamic, complex mixture of components, noise, and vibration. Polluted tunnel air was delivered to subjects in the nearby exposure facility while control animals received thoroughly filtered air from a tunnel-adjacent area. Because behavioral outcomes vary by sex, time of year, vendor, and numerous additional variables, we ran, in parallel, a laboratory control group^58,82^.

An important finding was that we observed no significant difference in litter size between TRAP, FA, and laboratory groups, which eliminated litter size as a potential explanatory variable for effects of near roadway exposure on pup growth and development. Yet, both roadside groups had significantly delayed growth and development of psychomotor reflexes, altered social interactions, and abnormal activity in an open field compared to laboratory controls. A potential explanation was that transport stress confounded our observations. However, we showed a complete absence of behavioral phenotypes resulting from the transport alone, strongly suggesting that adverse functional outcomes observed in the TRAP and FA groups were attributable to near roadway exposures. In both sexes of FA- and TRAP-exposed groups, we observed reduced isolation-induced 40-kHz pup ultrasonic vocalizations. Other atypical behaviors included juvenile social play behavior by the critical investigative parameter of anogenital sniffing and social play behavior of following/chasing. These data have direct translational implications as epidemiological studies directed at investigating ASD and NDDs have reported high levels of physical and developmental effects on health associated with the proximity of residence to heavily trafficked roads, using unique data sets from differing regional areas^21–24,28,75,83^. Follow-up studies will need to delineate the effects of noise and vibration during pregnancy from those of TRAP on offspring development and behavior. These findings add to our understanding of the risks posed to the developing nervous system by living in close proximity to roadways and support the need for further research delineating causal link(s) between exposure to TRAP and behavioral outcomes relevant to NDDs.

We observed a strong trend toward reduced overall social sniffing in the TRAP- and FA-exposed groups. Social sniffing is a key investigative behavior that initiates numerous types of juvenile social play events such as following/chasing, pinning, pouncing, and rough and tumble play. The TRAP- and FA-exposed juveniles exhibited these deficits without confounding motor deficits. In addition, we observed a trend to elevated self-grooming in males, a well-reported, standardized restricted, repetitive behavior in rodents^84^. Less social sniffing in dyadic interactions and elevated self-grooming are likely indicators of stress in both exposure groups.

In addition, others observed elevated self-grooming, repetitive behavior in mice. Similar findings of reduced reciprocal interactions following diesel exhaust exposure from prenatal embryonic day 0 to postnatal day 21 were recently reported in male mice^70^. Chang et al. also reported their diesel exhaust exposure caused increased repeated entries in the T-maze test of spontaneous alternation, a learning and memory assay with the embedded ability to capture restricted behavior. We observed high levels of repetitive motor behavior in the TRAP-exposed group, using a low-order motor stereotypy measure of grooming. Spontaneous alternation in T or Y maze, which may be mimicking higher order restricted, repetitive behaviors, are easily observable in mice^85–88^.

Surprisingly, we did not observe deficits on our two standard assays of learning and memory, contextual and cued fear conditioning and novel object recognition, due to TRAP exposure. We hypothesized this behavioral domain to have a robust phenotype given earlier literature. Early postnatal life exposure to concentrated ambient ultrafine particles (CAPS) increased preference for immediate reward, a more complex type of cognition that assesses impulsivity using a fixed-ratio waiting-for-reward paradigm, in mice^80,89^. The discounting of delayed rewards in preclinical models is considered to be analogous to impulsivity and delay of gratification in humans and is relevant to ADHD. Follow-up investigations revealed that early postnatal exposures to CAPS caused sexually dimorphic impairments in fixed interval performance on an operant training task, with greater sensitivity in males, while adult exposures caused deficits in females, indicating dysfunctional learning and reduced behavioral flexibility in CAPS-exposed mice. CAPS exposure also impaired short-term memory on the novel object recognition memory task in both sexes^38,80^. Collectively, these observations indicate dysfunctional learning and reduced behavioral flexibility in CAPS-exposed mice. The different results observed in the CAPS study versus our study may be due to (a) our tasks being limited to fear conditioning and novel object recognition because of limitations on the type of equipment that could be housed at the exposure facility, (b) that there is >20 million years of evolution that separate mouse and rat and there is likely species differences^90,91^, and/or (c) the variable intensities and concentrations of the exposures. Another limitation of our learning and memory data was that the laboratory control data were collected from cohorts other than those under study herein, thereby precluding direct comparisons of performance scores. We were, however, able to make observational assessments with the knowledge that the cohorts were all Sprague-Dawley rats of similar ages tested by the same experimenter using the same equipment following the standard experimental protocol and the laboratory controls were within our standard validation scores. In future studies, we plan on employing operant touchscreen testing, as performed by our laboratories in mice and rats^48,92,93^, which will allow for more direct comparisons of impulsivity via five choice serial reaction and continuous performance assays^94–98^. Other groups exposed rats in a highly trafficked location in Portugal to non-filtered air (NFA) during gestation and early life and found a significant decrease in object discrimination when compared to the group exposed to filtered air (FA), suggesting that the exposure to TRAP during the combined pre- and post-natal periods impaired short-term discriminative memory. Animals exposed during only pre- or post-natal period did not show impairment on this assay^99^, similar to our findings. Another group found that ambient concentrated PM_2.5_ exposure resulted in robust impairments in adult mice tested in the Barnes Maze, a hippocampal dependent spatial learning task. The PM_2.5_-exposed mice made more errors during training and took longer to reach the target during training trials and the memory retention test, indicating that chronic exposure to airborne fine particulate matter impaired hippocampal related learning and memory^100^.

Multiple groups have reported strong associations between prenatal exposure to TRAP and developmental delays and/or NDDs. Since epidemiology studies are associative, rigorous experiments that test preclinical models in highly controlled environments are warranted. This is particularly pertinent for studies of TRAP, since for decades research has focused on the detrimental effects of tobacco and asthma/allergy-related illnesses. In conclusion, we developed and functionally validated an innovative preclinical model that recapitulated human studies that have linked developmental exposure to TRAP, or proximity to TRAP, and increased risk of NDDs. This confirmation of TRAP as an environmental risk factor for NDDs provides a rationale for controlling and minimizing exposures during critical periods of neurodevelopment thereby reducing the incidence of NDDs and/or decreasing the severity of symptoms. This study sets the stage for future mechanistic investigations to determine the mechanisms by which this risk factor interacts with NDD genes of susceptibility. It will also inform our understanding of the molecular pathophysiology of NDDs, which will be useful for identifying developmental windows of vulnerability and possible novel intervention and/or therapeutic strategies.

## Supplementary information

Berg et al. Supplementary Information

## References

[1] Dall’Aglio L (2018). The role of epigenetic modifications in neurodevelopmental disorders: a systematic review. Neurosci. Biobehav. Rev..

[2] Dalley JW, Everitt BJ, Robbins TW (2011). Impulsivity, compulsivity, and top-down cognitive control. Neuron.

[3] Baio J (2018). Prevalence of autism spectrum disorder among children aged 8 years—autism and developmental disabilities monitoring network, 11 sites, United States, 2014. MMWR Surveill. Summ..

[4] Sahin M, Sur M (2015). Genes, circuits, and precision therapies for autism and related neurodevelopmental disorders. Science.

[5] Hallmayer J (2011). Genetic heritability and shared environmental factors among twin pairs with autism. Arch. Gen. Psychiatry.

[6] Zuk O, Hechter E, Sunyaev SR, Lander ES (2012). The mystery of missing heritability: genetic interactions create phantom heritability. Proc. Natl Acad. Sci. USA.

[7] Stamou M, Streifel KM, Goines PE, Lein PJ (2012). Neuronal connectivity as a convergent target of gene x environment interactions that confer risk for Autism Spectrum Disorders. Neurotoxicology Teratol..

[8] Pessah, I. N. & Lein P. J. in: *Autism: Current Theories and Evidence*. (edsZimmerman A.), 409–428 (Humana Press, Totowa, NJ, 2008).

[9] Hertz-Picciotto I, Delwiche L (2009). The rise in autism and the role of age at diagnosis. Epidemiology.

[10] Grether JK, Rosen NJ, Smith KS, Croen LA (2009). Investigation of shifts in autism reporting in the California Department of Developmental Services. J. Autism Dev. Disord..

[11] King M, Bearman P (2009). Diagnostic change and the increased prevalence of autism. Int. J. Epidemiol..

[12] Keil KP, Lein PJ (2016). DNA methylation: a mechanism linking environmental chemical exposures to risk of autism spectrum disorders?. Environ. Epigenet.

[13] Kalkbrenner AE (2010). Perinatal exposure to hazardous air pollutants and autism spectrum disorders at age 8. Epidemiology.

[14] Kalkbrenner AE (2015). Particulate matter exposure, prenatal and postnatal windows of susceptibility, and autism spectrum disorders. Epidemiology.

[15] Kalkbrenner AE (2018). Air Toxics in relation to autism diagnosis, phenotype, and severity in a U.S. family-based study. Environ. Health Perspect..

[16] McGuinn LA (2020). Early life exposure to air pollution and autism spectrum disorder: findings from a multisite case-control study. Epidemiology.

[17] Thygesen M (2020). Exposure to air pollution in early childhood and the association with attention-deficit hyperactivity disorder. Environ. Res..

[18] Ladd-Acosta C (2019). Epigenetic marks of prenatal air pollution exposure found in multiple tissues relevant for child health. Environ. Int.

[19] Volk HE (2014). Autism spectrum disorder: interaction of air pollution with the MET receptor tyrosine kinase gene. Epidemiology.

[20] Volk HE, Lurmann F, Penfold B, Hertz-Picciotto I, McConnell R (2013). Traffic-related air pollution, particulate matter, and autism. JAMA Psychiatry.

[21] Becerra TA, Wilhelm M, Olsen J, Cockburn M, Ritz B (2013). Ambient air pollution and autism in Los Angeles county, California. Environ. Health Perspect..

[22] Jung CR, Lin YT, Hwang BF (2013). Air pollution and newly diagnostic autism spectrum disorders: a population-based cohort study in Taiwan. PLoS ONE.

[23] von Ehrenstein OS, Aralis H, Cockburn M, Ritz B (2014). In utero exposure to toxic air pollutants and risk of childhood autism. Epidemiology.

[24] Windham GC, Zhang L, Gunier R, Croen LA, Grether JK (2006). Autism spectrum disorders in relation to distribution of hazardous air pollutants in the San Francisco bay area. Environ. Health Perspect..

[25] Herr CE (2011). Exposure to air pollution in critical prenatal time windows and IgE levels in newborns. Pediatr. Allergy Immunol..

[26] Kerin T (2018). Association between air pollution exposure, cognitive and adaptive function, and ASD severity among children with autism spectrum disorder. J. Autism Dev. Disord..

[27] Payne-Sturges DC (2019). Healthy air, healthy brains: advancing air pollution policy to protect children’s health. Am. J. Public Health.

[28] Volk HE, Hertz-Picciotto I, Delwiche L, Lurmann F, McConnell R (2011). Residential proximity to freeways and autism in the CHARGE study. Environ. Health Perspect..

[29] Chun H, Leung C, Wen SW, McDonald J, Shin HH (2020). Maternal exposure to air pollution and risk of autism in children: a systematic review and meta-analysis. Environ. Pollut..

[30] Goodrich AJ (2018). Joint effects of prenatal air pollutant exposure and maternal folic acid supplementation on risk of autism spectrum disorder. Autism Res..

[31] Chandrakumar A, Willem’t Jong G (2019). Maternal exposure to air pollution during pregnancy and autism spectrum disorder in offspring. JAMA Pediatr..

[32] Pagalan L (2019). Association of prenatal exposure to air pollution with autism spectrum disorder. JAMA Pediatr..

[33] Pagalan L, Brauer M, Lanphear B (2019). Maternal exposure to air pollution during pregnancy and autism spectrum disorder in offspring-reply. JAMA Pediatr..

[34] Bein KJ, Wexler AS (2014). A high-efficiency, low-biased method for extracting particulate matter from filter and impactor substrates. Atmos. Environ..

[35] Plummer L, Ham W, Kleeman M, Wexler A, Pinkerton K (2012). Influence of season and location on pulmonary response to California’s San Joaquin Valley airborne particulate matter. J. Toxicol. Environ. Health Part A.

[36] Zhang KM, Wexler AS (2004). Evolution of particle number distribution near roadways. Part I: analysis of aerosol dynamics and its implications for engine emission measurement. Atmos. Environ..

[37] Zhang KM (2005). Evolution of particle number distribution near roadways. Part III: Traffic analysis and on-road size resolved particulate emission factors. Atmos. Environ..

[38] Cory-Slechta DA, Allen JL, Conrad K, Marvin E, Sobolewski M (2018). Developmental exposure to low level ambient ultrafine particle air pollution and cognitive dysfunction. Neurotoxicology.

[39] Sobolewski M (2018). Developmental exposures to ultrafine particle air pollution reduces early testosterone levels and adult male social novelty preference: Risk for children’s sex-biased neurobehavioral disorders. Neurotoxicology.

[40] Carosino CM (2015). Allergic airway inflammation is differentially exacerbated by daytime and nighttime ultrafine and submicron fine ambient particles: heme oxygenase-1 as an indicator of PM-mediated allergic inflammation. J. Toxicol. Environ. Health, Part A.

[41] Plummer LE (2015). Pulmonary inflammatory effects of source-oriented particulate matter from California’s San Joaquin Valley. Atmos. Environ..

[42] Papapostolou V (2012). Development and characterization of an exposure generation system to investigate the health effects of particles from fresh and aged traffic emissions. Air Qual., Atmosphere Health.

[43] Bein K. J. et al. Roadway tunnels as real-time exposure systems: a case study. *Environ Sci. Technol.* (2020).

[44] Hertz-Picciotto I (2006). The CHARGE study: an epidemiologic investigation of genetic and environmental factors contributing to autism. Environ. health Perspect..

[45] Patten KT (2020). Effects of early life exposure to traffic-related air pollution on brain development in juvenile Sprague-Dawley rats. Transl. psychiatry.

[46] Hood R. D. *Handbook of Developmental Toxicology* (CRC Press, USA, 1996).

[47] Lazic SE, Essioux L (2013). Improving basic and translational science by accounting for litter-to-litter variation in animal models. BMC Neurosci..

[48] Berg EL (2020). Translational outcomes in a full gene deletion of ubiquitin protein ligase E3A rat model of Angelman syndrome. Transl. Psychiatry.

[49] Adhikari A (2019). Cognitive deficits in the Snord116 deletion mouse model for Prader-Willi syndrome. Neurobiol. Learn. Mem..

[50] Fox WM (1965). Reflex-ontogeny and behavioural development of the mouse. Anim. Behav..

[51] Hofer, M. A., Shair, H. N. & Brunelli S. A. Ultrasonic vocalizations in rat and mouse pups. Curr. Protoc. Neurosci. **Chapter 8:** Unit 8.14 (2002). 10.1002/0471142301.ns0814s17 18428567

[52] Wohr M, Schwarting RK (2008). Maternal care, isolation-induced infant ultrasonic calling, and their relations to adult anxiety-related behavior in the rat. Behav. Neurosci..

[53] Brudzynski, S. *Handbook of Ultrasonic Vocalization: A Window Into the Emotional Brain*, vol. 25 (Academic Press, 2018).

[54] Berg EL (2018). Developmental social communication deficits in the Shank3 rat model of Phelan-Mcdermid syndrome and autism spectrum disorder. Autism Res..

[55] Copping NA (2017). Neuronal overexpression of Ube3a isoform 2 causes behavioral impairments and neuroanatomical pathology relevant to 15q11.2-q13.3 duplication syndrome. Hum. Mol. Genet..

[56] Gulinello T (2019). Rigor and reproducibility in rodent behavioral research. Neurobiol. Learn. Mem..

[57] Gompers AL (2017). Germline Chd8 haploinsufficiency alters brain development in mouse. Nat. Neurosci..

[58] Sukoff Rizzo SJ, Silverman JL (2016). Methodological considerations for optimizing and validating behavioral assays. Curr. Protoc. Mouse Biol..

[59] Hofer MA, Shair HN (1992). Ultrasonic vocalization by rat pups during recovery from deep hypothermia. Dev. Psychobiol..

[60] Shair HN, Brunelli SA, Masmela JR, Boone E, Hofer MA (2003). Social, thermal, and temporal influences on isolation-induced and maternally potentiated ultrasonic vocalizations of rat pups. Dev. Psychobiol..

[61] Allin JT, Banks EM (1971). Effects of temperature on ultrasound production by infant albino rats. Dev. Psychobiol..

[62] Oswalt GL, Meier GW (1975). Olfactory, thermal, and tactual influences on infantile ultrasonic vocalization in rats. Dev. Psychobiol..

[63] Raza S (2015). Effects of prenatal exposure to valproic acid on the development of juvenile-typical social play in rats. Behav. Pharm..

[64] Thor DH, Holloway WR (1984). Social play in juvenile rats: a decade of methodological and experimental research. Neurosci. Biobehav. Rev..

[65] Vanderschuren LJ, Niesink RJ, Van Ree JM (1997). The neurobiology of social play behavior in rats. Neurosci. Biobehav. Rev..

[66] Ku KM, Weir RK, Silverman JL, Berman RF, Bauman MD (2016). Behavioral phenotyping of juvenile long-evans and Sprague-Dawley rats: implications for preclinical models of autism spectrum disorders. PLoS ONE.

[67] Panksepp J (1981). The ontogeny of play in rats. Dev. Psychobiol..

[68] Panksepp J, Beatty WW (1980). Social deprivation and play in rats. Behav. Neural Biol..

[69] Costa LG, Chang YC, Cole TB (2017). Developmental neurotoxicity of traffic-related air pollution: focus on autism. Curr. Environ. Health Rep..

[70] Chang YC, Cole TB, Costa LG (2018). Prenatal and early-life diesel exhaust exposure causes autism-like behavioral changes in mice. Part Fibre Toxicol..

[71] Lyall K (2017). The changing epidemiology of autism spectrum disorders. Annu. Rev. Public Health.

[72] Costa LG (2017). Neurotoxicity of traffic-related air pollution. Neurotoxicology.

[73] Kim D (2017). The joint effect of air pollution exposure and copy number variation on risk for autism. Autism Res..

[74] Block ML (2012). The outdoor air pollution and brain health workshop. Neurotoxicology.

[75] Hansen CA, Barnett AG, Pritchard G (2008). The effect of ambient air pollution during early pregnancy on fetal ultrasonic measurements during mid-pregnancy. Environ. Health Perspect..

[76] Allen JL (2014). Early postnatal exposure to ultrafine particulate matter air pollution: persistent ventriculomegaly, neurochemical disruption, and glial activation preferentially in male mice. Environ. Health Perspect..

[77] Allen JL (2014). Developmental exposure to concentrated ambient ultrafine particulate matter air pollution in mice results in persistent and sex-dependent behavioral neurotoxicity and glial activation. Toxicol. Sci..

[78] Zhao Y. et al. Field evaluation of the versatile aerosol concentration enrichment system (VACES) particle concentrator coupled to the rapid single-particle mass spectrometer (RSMS-3). *J. Geophys. Res. Atmos.*10.1029/2004JD004644 (2005).

[79] Breysse PN (2013). US EPA particulate matter research centers: summary of research results for 2005–2011. Air Qual. Atmos. Health.

[80] Allen JL (2017). Developmental neurotoxicity of inhaled ambient ultrafine particle air pollution: Parallels with neuropathological and behavioral features of autism and other neurodevelopmental disorders. Neurotoxicology.

[81] Allen JL (2014). Consequences of developmental exposure to concentrated ambient ultrafine particle air pollution combined with the adult paraquat and maneb model of the Parkinson’s disease phenotype in male mice. Neurotoxicology.

[82] Silverman JL, Yang M, Lord C, Crawley JN (2010). Behavioural phenotyping assays for mouse models of autism. Nat. Rev. Neurosci..

[83] Currie J, Neidell M, Schmieder JF (2009). Air pollution and infant health: Lessons from New Jersey. J. Health Econ..

[84] Kalueff AV (2016). Neurobiology of rodent self-grooming and its value for translational neuroscience. Nat. Rev. Neurosci..

[85] Sukoff Rizzo SJ (2018). Assessing healthspan and lifespan measures in aging mice: optimization of testing protocols, replicability, and rater reliability. Curr. Protoc. Mouse Biol..

[86] Sukoff Rizzo SJ, Crawley JN (2017). Behavioral phenotyping assays for genetic mouse models of neurodevelopmental, neurodegenerative, and psychiatric disorders. Annu Rev. Anim. Biosci..

[87] Sukoff Rizzo SJ (2014). Behavioral characterization of striatal-enriched protein tyrosine phosphatase (STEP) knockout mice. Genes Brain Behav..

[88] Sukoff Rizzo SJ, McTighe S, McKinzie DL (2019). Genetic background and sex: impact on generalizability of research findings in pharmacology studies. Handb. Exp. Pharm..

[89] Allen JL (2013). Developmental exposure to concentrated ambient particles and preference for immediate reward in mice. Environ. Health Perspect..

[90] Iannaccone PM, Jacob HJ (2009). Rats!. Dis. Model. Mech..

[91] Parker CC (2014). Rats are the smart choice: rationale for a renewed focus on rats in behavioral genetics. Neuropharmacology.

[92] Copping NA (2017). Touchscreen learning deficits and normal social approach behavior in the Shank3B model of Phelan-McDermid Syndrome and autism. Neuroscience.

[93] Petkova SP (2020). Cyclin D2-knock-out mice with attenuated dentate gyrus neurogenesis have robust deficits in long-term memory formation. Sci. Rep..

[94] Bhakta SG, Young JW (2017). The 5 choice continuous performance test (5C-CPT): a novel tool to assess cognitive control across species. J. Neurosci. methods.

[95] Cope ZA, Young JW (2017). The five-choice continuous performance task (5C-CPT): a cross-species relevant paradigm for assessment of vigilance and response inhibition in rodents. Curr. Protoc. Neurosci..

[96] Lustig C, Kozak R, Sarter M, Young JW, Robbins TW (2013). CNTRICS final animal model task selection: control of attention. Neurosci. Biobehav. Rev..

[97] McKenna BS, Young JW, Dawes SE, Asgaard GL, Eyler LT (2013). Bridging the bench to bedside gap: validation of a reverse-translated rodent continuous performance test using functional magnetic resonance imaging. Psychiatry Res.

[98] Cope ZA (2016). Premature responses in the five-choice serial reaction time task reflect rodents’ temporal strategies: evidence from no-light and pharmacological challenges. Psychopharmacology.

[99] Zanchi AC (2010). Pre and post-natal exposure to ambient level of air pollution impairs memory of rats: the role of oxidative stress. Inhal. Toxicol..

[100] Fonken LK (2011). Air pollution impairs cognition, provokes depressive-like behaviors and alters hippocampal cytokine expression and morphology. Mol. Psychiatry.

