# Supplementary material for: Developmental exposure to near roadway pollution produces behavioral phenotypes relevant to neurodevelopmental disorders in juvenile rats
Source: Transl Psychiatry. 2020 Aug 17;10:289. doi: 10.1038/s41398-020-00978-0 (PMC7431542; doi:10.1038/s41398-020-00978-0)
Supplement: Supplementary file 1 — Berg et al. Supplementary Information [file 41398_2020_978_MOESM1_ESM.docx]

**Supplementary Information**

(8 pages, 4 figures, 3 tables)

**Developmental Exposure to Near Roadway Pollution Produces Behavioral Phenotypes Relevant to Neurodevelopmental Disorders in Juvenile Rats**

Elizabeth L. Berg^1^, Lauren R. Pedersen^1^, Michael C. Pride^1^, Stela P. Petkova^1^, Kelley T. Patten^2^, Anthony E. Valenzuela^2^, Christopher Wallis^3^, Keith J. Bein^3^, Anthony Wexler^3^, Pamela J. Lein^2^, and Jill L. Silverman^1*^

^1^MIND Institute and Department of Psychiatry and Behavioral Sciences, University of California Davis School of Medicine, Sacramento, CA, USA.

^2^Department of Molecular Biosciences, University of California Davis School of Veterinary Medicine, Davis, CA, USA.

^3^Air Quality Research Center, University of California Davis, Davis, CA, USA.

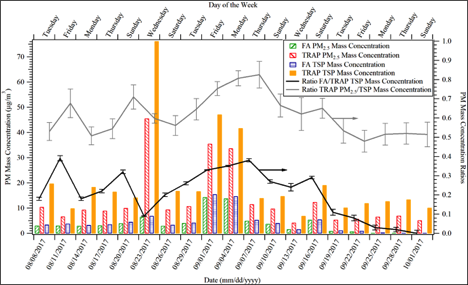


**Figure S1. Bars/left axis: temporal trends in 24-hour PM2.5 and Total Suspended Particulate (TSP) mass concentrations measured immediately upstream of the Filtered Air (FA) and Traffic-Related Air Pollution (TRAP) exposure chambers at the Facility for Roadway Air Pollution Exposure for the study duration.** Lines/markers/right axis: temporal trends in the ratio of FA to TRAP TSP mass concentrations (black line) and ratio of TRAP PM2.5 to TSP (gray line). Filter-based PM samplers (1) owned and maintained by the Interagency Monitoring of Protected Visual Environments (IMPROVE) were deployed in this study to collect 24-hour continuous PM samples every third day for the study duration. PM mass concentrations were determined from gravimetric analysis of the collected filter samples according to the handling, storage, measurement and QA/QC protocols of the IMPROVE Particle Monitoring Network (2). Random errors associated with gravimetric analysis of the filter samples are estimated to be ± 3 mg per measurement, which equates to ± 0.1 mg/m^3^ for the concentration calculations. Propagation of these uncertainties in the calculation of mass concentration ratios are included in the lines of the plot.

**Fig. S2. Exposure to transportation at ~GD14 did not alter juvenile reciprocal social interactions on several key parameters.** The transportation did not account for the findings observed in the roadside exposure groups in social play including A) levels of exploration during the social interaction assay, B) time social sniffing all body parts, C) time spent anogenital sniffing, and D) time spent following or chasing the stimulus animals. E) There was no effect of the transport on offspring measured by the time spent self-grooming compared to lab controls from dams that did not undergo the transport event.

**Fig. S3. Performance of laboratory-reared Sprague-Dawley rats in the test of novel object recognition.** Following the same testing protocol employed at the tunnel-adjacent exposure facility, Sprague-Dawley rat A) males (N=16) and B) females (N=15) reared and tested under UC Davis laboratory conditions spent significantly more time sniffing the novel object compared to the familiar object. **p* < 0.05, paired *t*-test.

**Fig. S4. Performance by laboratory-reared Sprague-Dawley rats within the contextual and cued fear conditioning paradigm.** Following the same testing protocol employed at the tunnel-adjacent exposure facility, A) male Sprague-Dawley rats (N=26) reared and tested under UC Davis laboratory conditions exhibited little to no freezing pre-training and pre-cue, spent approximately 50% of the time freezing post-training and during the cue in a novel context, and 30% time freezing in the training context. B) Female Sprague-Dawley rats (N=23) reared and tested in a UC Davis laboratory exhibited little to no freezing pre-training and pre-cue, spent approximately 40% of the time freezing post-training and during the cue in a novel context, and 15% time freezing in the training context.
